# Preparation, structural property, and antioxidant activities of a novel pectin polysaccharide from the flowers of *Hibiscus syriacus* Linn.

**DOI:** 10.3389/fnut.2024.1524846

**Published:** 2025-01-07

**Authors:** Jialong Chen, Chaojun Ye, Lu Zhang, Zhiliang Xie, Jianjun Zhu, Zhi Zhang

**Affiliations:** ^1^Wenzhou Academy of Agricultural Sciences/Key Laboratory of Crop Breeding in South Zhejiang, Wenzhou Vocational College of Science and Technology, Wenzhou, China; ^2^College of Landscape and Architecture, Zhejiang Agriculture and Forestry University, Hangzhou, China; ^3^Prefabricated Dish Industry Development Research Institute, Zhejiang Dong Fang Polytechnic, Wenzhou, China

**Keywords:** *Hibiscus syriacus* Linn., pectin polysaccharide, structural characterization, antioxidant activity, oxidative stress, Nrf2/HO-1 pathway

## Abstract

**Introduction:**

Oxidative stress, triggered by an imbalance between reactive oxygen species (ROS) production and cellular antioxidant defense mechanisms, is implicated in various pathological conditions. Plant-derived polysaccharides have gained significant attention as potential natural antioxidants due to their biocompatibility, biodegradability, and structural versatility.

**Methods:**

This study focuses on the purification, structural characterization, and antioxidant activities of a novel pectin polysaccharide (HFPS) isolated from the flowers of *Hibiscus syriacus* Linn. HFPS was purified using anion-exchange chromatography. Its chemical composition, monosaccharide profile, molecular weight distribution, and structural properties were elucidated through various analytical techniques.

**Results:**

HFPS exhibited a predominant acidic monosaccharide composition, with galacturonic acid as the major constituent, indicating its pectin nature. The free radical scavenging activity of HFPS against ABTS·, DPPḤ, and ·OH radicals was evaluated, demonstrating a positive correlation between its antioxidant capacity and concentration. Furthermore, HFPS effectively protected HepG2 cells against H_2_O_2_-induced oxidative stress by reducing ROS accumulation, modulating redox-related enzymes (Superoxide dismutase, Catalase, Glutathione peroxidase), and alleviating oxidative damage. Notably, HFPS upregulated the expression of antioxidant-related genes, including *B-cell lymphoma-2 (Bcl-2), heme oxygenase 1 (HO-1), NAD(P)H quinone oxidoreductase 1 (NQO1), and nuclear factor erythroid 2-related factor 2 (Nrf2)*, while downregulating pro-apoptotic genes like Bcl-2-associated X protein (Bax) and Caspase-3. These findings suggest that HFPS exerts its antioxidant effects by modulating the Nrf2/HO-1 pathway and redox homeostasis.

**Discussion:**

This study contributes to the understanding of the structure-function relationships and antioxidant mechanisms of HFPS, highlighting its potential applications as a natural antioxidant in various fields.

## 1 Introduction

Oxidative stress, characterized by an imbalance between the production of ROS and the cellular antioxidant defense mechanisms, leading to associated functional cell damage or apoptosis, exacerbating the inflammatory response, and has been implicated in various pathological conditions, including cardiovascular diseases, neurodegenerative disorders, and cancer (1, 2). Excessive levels of ROS can induce oxidative damage to biomolecules, such as lipids, proteins, and nucleic acids, leading to cellular dysfunction and tissue injury (3). This underscores the growing interest in exploring natural antioxidants as potential therapeutic agents to counteract oxidative stress and its associated deleterious effects.

Plant-derived polysaccharides have gained considerable attention due to their diverse bioactivities, including antioxidant, anti-inflammatory, and immunomodulatory properties (4, 5). These natural biopolymers possess several advantages over synthetic antioxidants, such as biocompatibility, biodegradability, and structural versatility, making them attractive candidates for functional food ingredients and therapeutic applications (6). Various studies have investigated the antioxidant potential of polysaccharides extracted from various plant sources, including fruits, vegetables, and medicinal herbs (7, 8). Despite these promising findings, the mechanistic understanding of the antioxidant activities of plant polysaccharides remains limited. Several studies have suggested that the antioxidant effects of polysaccharides may be attributed to their ability to scavenge free radicals, chelate metal ions, and modulate the expression of antioxidant enzymes and related signaling pathways, such as Nrf2/HO-1 and PI3K/Akt, which serve as the major regulatory pathways of intracellular defense against oxidative stress and are considered an ideal target for alleviating endothelial cell injury (9, 10). However, the specific mechanisms underlying these activities often vary depending on the structural characteristics and chemical composition of the polysaccharides, necessitating further investigation. In this context, comprehensive characterization of the chemical and structural features of plant polysaccharides is essential for elucidating their structure–function relationships and antioxidant mechanisms.

Exploring novel polysaccharides from diverse plant sources is crucial for identifying potential antioxidant agents. *Hibiscus syriacus* Linn., commonly known as the Chinese hibiscus or shoeblack plant, is an ornamental plant widely cultivated for its vibrant flowers. Beyond its decorative value, various parts of this plant, including the flowers, have been utilized in traditional medicine to treat various ailments (11). Despite its widespread cultivation and traditional usage, the bioactive components present in *Hibiscus syriacus* Linn. flowers, particularly its polysaccharide fraction, remain largely unexplored. Preliminary studies have indicated the presence of polysaccharides in the flowers of this plant, which exhibit potential antioxidant properties (12). However, comprehensive investigations into the structural characterization, antioxidant mechanisms, and biological activities of these polysaccharides are still lacking.

In the present study, we focus on the purification and characterization of a novel alkali-extracted pectin polysaccharide isolated from the flowers of *Hibiscus syriacus* Linn. (HFPS). By elucidating its chemical composition, monosaccharide profile, and structural properties, we aim to establish a foundation for understanding its antioxidant potential and underlying mechanisms. Furthermore, the evaluation of HFPS’s free radical scavenging activity and protective effects against oxidative stress in cell models can provide insights into its potential applications as a natural antioxidant in various fields, such as functional foods, pharmaceuticals, and biomaterials.

## 2 Materials and methods

### 2.1 Materials and chemicals

The dried *Hibiscus syriacus* Linn. flower was purchased from Bozhou Haoyitang Biotechnology Co., Ltd. (Bozhou, China). The HepG2 cells were obtained from the cell bank of the Chinese Academy of Science (Shanghai, China). The DEAE Sepharose Fast Flow gel was purchased from GE Co. (St. Louis, MO, USA). The DMEM medium was purchased from HyClone Co. (Los Angeles, USA). The 3-(4,5-dimethyl-2-thiazolyl)-2,5-diphenyl-2H-tetrazolium bromide (MTT) was purchased from Solarbio Co. (Beijing, China). Malondialdehyde (MDA), Superoxide dismutase (SOD), Catalase (CAT), and Glutathione peroxidase (GSH-Px) detecting ELISA kits were obtained from Nanjing Jiancheng Bioengineering Institute (Nanjing, China). All other chemicals and solvents were of analytical grade.

### 2.2 Pectin polysaccharide extraction and purification

The extraction procedure was carried out according to the method described by Zhang et al. (13). The dried *Hibiscus syriacus* Linn. flower powder was mixed with deionized water at a ratio of 30:1 (w/w). The pH of the mixture was adjusted to 12 by adding 1.0 M sodium hydroxide (NaOH) solution. The alkalinized mixture was then extracted at 70°C for 90 min under continuous magnetic stirring in a 1 L conical flask. After extraction, the mixture was cooled to room temperature and centrifuged at 9,600×*g* for 10 min at 4°C to separate the supernatant containing the solubilized pectin. The supernatant was collected, and pectin precipitation was induced by adding three volumes of 95% ethanol while maintaining the temperature at 4°C overnight. The precipitated pectin was recovered by centrifugation at 9,600×*g* for 10 min. Subsequently, the crude pectin was redissolved in an appropriate volume of deionized water and subjected to dialysis against deionized water using a dialysis bag with a 3.5 kDa Mw cut-off. The dialysis process involved replacing the external water every 12 h until the pH of the dialysate became neutral. Finally, the dialyzed pectin solution was freeze-dried to obtain the preliminary purified alkaline-extracted HFPS, which was stored at 4°C until further analysis and characterization.

The preliminary purified polysaccharide sample was dissolved in an appropriate amount of ddH_2_O and centrifuged at 8,000 rpm for 10 min to remove insoluble particles, followed by filtration through a 0.45 μm membrane. The filtrate was loaded onto a pre-equilibrated DEAE Sepharose FF column, with a loading volume of 30% of the column bed volume. Subsequently, the column was sequentially eluted with NaCl solutions of different concentrations (0, 0.2 M, 0.5 M, and 1.0 M), using two-column bed volumes for each concentration at a flow rate of 15 mL/min. Fractions were collected in 100 tubes, with 10 mL per tube. The polysaccharide content in the eluates was monitored at 630 nm using the anthrone-sulfuric acid method, and the elution profile was plotted with tube number on the x-axis and absorbance on the y-axis. The combined fractions from the 0.2 M NaCl elution were concentrated under reduced pressure and dialyzed by a 3.5 kDa Mw cut-off dialysis membrane. The dialyzed sample was then lyophilized to obtain the purified HFPS fraction.

### 2.3 Chemical composition

The total sugar content was determined using the phenol-sulfuric acid assay with glucose as the standard (14), and the absorbance of the sample was measured at 490 nm. The uronic acid content was measured by the meta-hydroxydiphenyl method with galacturonic acid as standard (15), and the absorbance of the sample was measured at 450 nm. The protein content was quantified employing the well-established Bradford assay with bovine serum albumin as the standard (14), and the absorbance of the sample was measured at 595 nm. The polyphenol content was determined using the forintol method, with gallic acid as a standard (16), and the absorbance of the sample was measured at 760 nm.

### 2.4 Monosaccharide composition

A sample (5 mg) was hydrolyzed with 1 mL of 2 M trifluoroacetic acid (TFA) at 121°C for 2 h, followed by drying under a nitrogen stream. The residue was washed repeatedly with 3 mL of methanol and dried. Subsequently, 5 mL of ddH_2_O was added to dissolve the sample, and the solution was transferred to a vial. For derivatization, 0.2 mL of the hydrolyzed polysaccharide solution was mixed thoroughly with 0.2 mL of 0.5 M NaOH and 0.5 mL of 0.5 M 1-phenyl-3-methyl-5-pyrazolone (PMP) in methanol. The mixture was incubated at 70°C for 1 h. After the reaction, 0.2 mL of 0.5 M HCl was added to adjust the pH. Excess PMP was removed by extracting three times with 1 mL of chloroform. The aqueous layer was collected, diluted to 1 mL with water, and 0.3 mL was injected for analysis. The monosaccharide composition was determined using a ThermoU3000 liquid chromatography system (UltiMate 3000, Thermo Scientific, Waltham, MA, USA) equipped with a ZORBAX Eclipse XDB-C18 column. Isocratic elution was performed with a mobile phase of acetonitrile: phosphate buffer (17:83, v:v, pH 6.8) at a flow rate of 0.8 mL/min and a column temperature of 30°C. Detection was carried out at 250 nm with an injection volume of 10 μL. Rhamnose (Rha), arabinose (Ara), galactose (Gal), glucose (Glc), xylose (Xyl), mannose (Man), fucose (Fuc), galacturonic acid (GalA), glucuronic acid (GlcA), glucosamine hydrochloride (GlcN), and galactosamine hydrochloride (GalN) were selected as monosaccharide standards (4).

### 2.5 Mw distribution

The polysaccharide sample (5 mg) was dissolved in 1 mL of 0.05 M NaCl solution, centrifuged for 10 min (8,000×*g*), and the supernatant was collected and filtered through a 0.22 μm membrane. The filtrate was then transferred to a 2 mL vial for analysis. The Mw distribution of the sample was determined using a Waters 1,515 high-performance liquid chromatography (HPLC) system (Waters, Milford, MA, USA) equipped with a Waters 2,410 differential refractive index detector, three serially connected polymer-based aqueous size-exclusion chromatography (SEC) columns (8 × 300 mm) (Ohpak SB-803 HQ, Ohpak SB-804 HQ, and Ohpak SB-805 HQ), and a Waters 2,707 autosampler. The mobile phase was 0.05 M NaCl solution, with a flow rate of 0.65 mL/min, a column temperature of 40°C, and an injection volume of 30 μL. To generate a standard curve, the column was calibrated using dextran standards ranging from 1 to 760 kDa in Mw (15).

### 2.6 FT-IR spectra

The structural characteristics and functional groups of HFPS were investigated using an FT-IR spectrometer (Thermo ESCALAB 250, Waltham, MA, USA). The sample powder was dried, mixed with potassium bromide (KBr) powder, and compressed into a pellet using a hydraulic press. The pellet was then scanned over a frequency range of 4,000–500 cm^−1^ with a resolution of 4 cm^−1^. The resultant spectra were smoothed to remove noise, and baseline correction and peak intensity normalization were performed (17).

### 2.7 TG analysis

The thermal stability and degradation temperature of HFPS were evaluated using a simultaneous thermal analyzer (NETZSCH STA 449 F3, Selb, Free State of Bavaria, Germany). Under a nitrogen atmosphere, approximately 10 mg of the sample was heated at a rate of 10°C/min, and the weight loss was observed over the temperature range of 30–800°C (17).

### 2.8 XRD analysis

The crystalline property of HFPS was analyzed using an X-ray diffractometer (Pert3 Powder5, PANalytica, Almelo, The Netherlands) with Cu Kα radiation. The sample powder was scanned over a diffraction angle (2θ) range of 5°–90° with a step size of 0.02° (17).

### 2.9 SEM analysis

The surface morphology of HFPS was observed using a scanning electron microscope (Zeiss Sigma 300, UK). The sample was fixed and coated with gold under vacuum conditions. Micrographs were obtained at an accelerating voltage of 4 kV and magnifications of 20 k× and 1 k× (13).

### 2.10 NMR analysis

Approximately 10 mg of HFPS was dissolved in 1 mL of 99.98% deuterium oxide (D_2_O), and the solution was subjected to repeated lyophilization three times. The ^1^H and ^13^C NMR spectrum was recorded using a Bruker 400 MHz NMR spectrometer (Bruker, Billerica, MA, USA) (15).

### 2.11 Radical scavenging assay

The 2,2-diphenyl-1-picrylhydrazyl (DPPḤ), 2,2′-azino-bis(3-ethylbenzothiazoline-6-sulfonic acid) (ABTS·), and hydroxyl radical (·OH) scavenging activities of HFPS were evaluated according to the method described by Zhu et al. (4, 18).

### 2.12 Evaluation of cell models against oxidative stress

#### 2.12.1 Cell culture

HepG2 cells were cultured in DMEM medium supplemented with 10% fetal bovine serum (HFPS), 100 U/mL penicillin, and 100 U/mL streptomycin at 37°C in a humidified atmosphere of 5% CO_2_. Cells were passaged at 70–80% confluency and subcultured for three passages before experiments using logarithmic growth phase cells.

#### 2.12.2 Cell viability

HepG2 cells were seeded in 96-well plates at 2 × 10^5^ cells/well for 24 h. After removing the medium, cells were treated with various HFPS concentrations (25, 50, 100, 250, 500, and 1,000 μg/mL) for 36 h. The supernatant was discarded, and 100 μL of MTT solution (0.5 mg/mL) was added to each well, followed by 4 h incubation. The medium was removed, 100 μL of dimethyl sulfoxide was added, and absorbance was measured at 490 nm.

#### 2.12.3 MDA level, SOD, GSH-Px, and CAT activities

The experiment was divided into five groups, including Normal, Model, and three HFPS-treated groups. HepG2 cells (2 × 10^5^ cells/well) were seeded in 96-well plates for 24 h. The Model group was cultured in serum-free DMEM for 24 h, then treated with 2 μM H_2_O_2_ for 4 h. The HFPS-treated groups were treated with 100 μg/mL HFPS (L-HFPS), 250 μg/mL HFPS (M-HFPS), or 500 μg/mL HFPS (H-HFPS) for 24 h, followed by 2 μM H_2_O_2_ for 4 h. After centrifugation, MDA level, SOD, GSH-Px, and CAT activities in the supernatants were determined using commercial assay kits according to the manufacturer’s instructions.

#### 2.12.4 qPCR assay

Normal, Model, and HFPS-treated groups were established as described above using 5 × 10^5^ cells/mL in 60 mm^2^ dishes. Total RNA was extracted using TRIzol reagent (Service, Wuhan, China), and cDNA was synthesized by reverse transcription. qPCR was performed to evaluate mRNA levels of *Bax, Bcl-2*, *Caspase-3*, *HO-1*, *NQO1*, and *Nrf2*, using *GAPDH* as the housekeeping gene (primer sequences in Table 1). Reverse transcription and qPCR parameters followed the manufacturer’s protocols.

**Table 1:** Sequences of primer.

Primer name		Primer sequence
Bax	5′	TTGCTACAGGGTTTCATCCAG
	3′	TGTTGTTGTCCAGTTCATCG
Bcl-2	5′	GGGGCTACGAGTGGGATACT
	3′	GACGGTAGCGACGAGAGAAG
Casepase-3	5′	ACTGGAATGTCAGCTCGCAA
	3′	TTTTCAGGTCCACAGGTCCG
HO-1	5′	TGCCAGTGCCACCAAGTTCAAG
	3′	TGTTGAGCAGGAACGCAGTCTTG
NQO1	5′	AACCAACAGAGCCAATC
	3′	CCTCCATCCTTTCCTC
Nrf2	5′	ACGGTATGCAACAGGACATTGAGC
	3′	TTGGCTTCTGGACTTGGAACCATG
GAPDH	5′	GGGTCATCATCTCTGCACCT
	3′	GGTCATAAGTCCCTCCACGA

#### 2.12.5 Western blotting assay

Normal, Model, and HFPS-treated groups were established as described above using 5 × 10^5^ cells/mL in 60 mm^2^ dishes. After treatments, cells were lysed with RIPA buffer, and protein concentrations were determined by BCA assay. Proteins were denatured, separated by SDS-PAGE, transferred to PVDF membranes, and probed with primary antibodies overnight at 4°C. After washing, membranes were incubated with secondary antibodies at 37°C for 1 h. Protein bands were visualized using a chemiluminescence imaging system and quantified with Image Lab software version 6.0.

### 2.13 Statistical analysis

All experiments were conducted in triplicate. Statistical analysis was performed using one-way analysis of variance (ANOVA) with GraphPad Prism 8.0 (GraphPad Software Inc., San Diego, CA, USA), and the data are presented as mean ± standard deviation (SD). Differences were considered statistically significant at *p* < 0.05.

## 3 Results and discussion

### 3.1 Purification and chemical composition analysis

**Figure 1:**
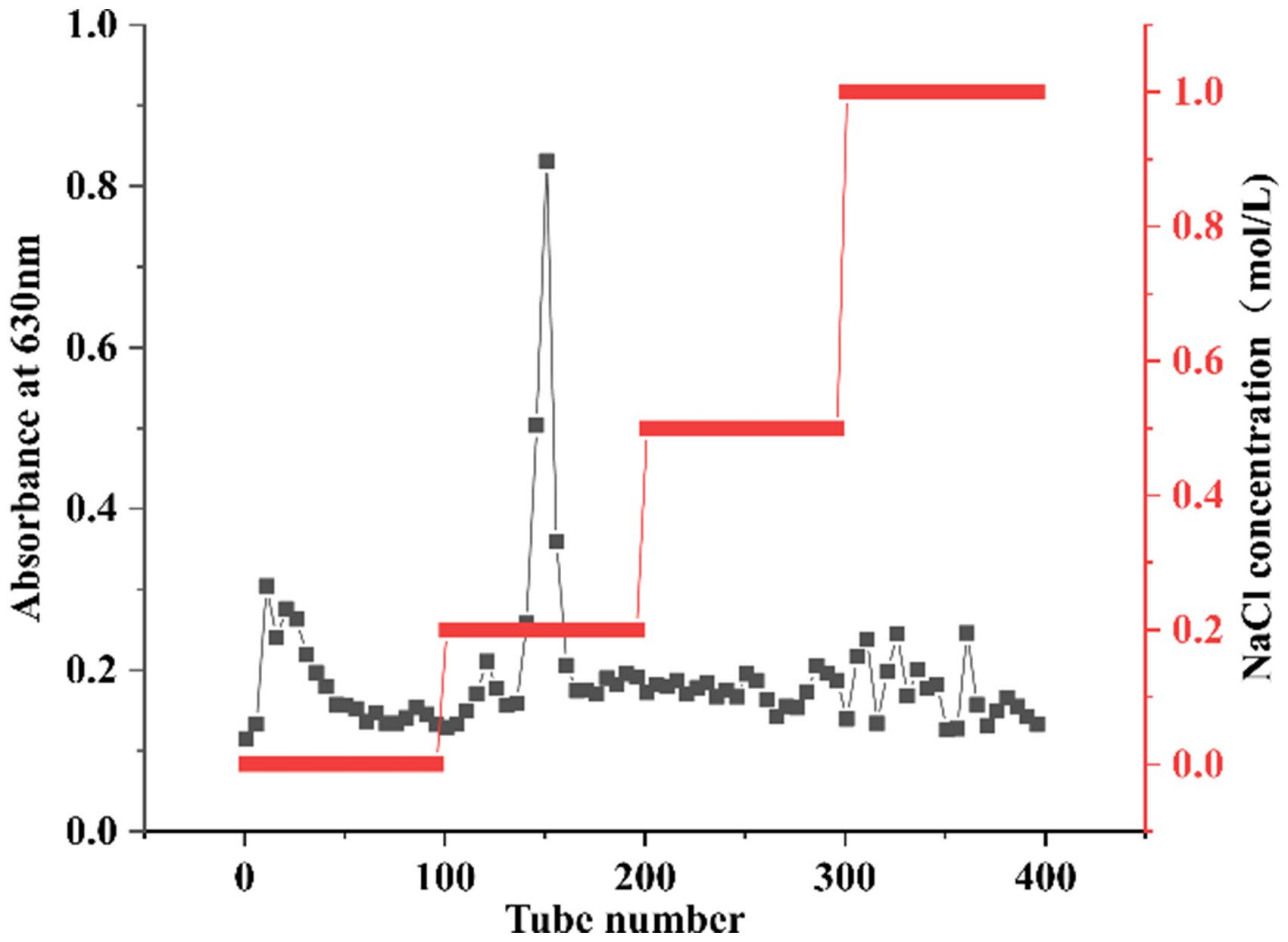
Elution curve of HFPS eluted with NaCl solutions of different concentrations (0, 0.2, 0.5, 1 M).

Due to the different charges of different polysaccharides, crude polysaccharides were eluted with NaCl solutions of different concentrations. As shown in Figure 1, the main polysaccharide component was eluted with 0.2 M NaCl, yielding the purified HFPS (hereafter referred to as HFPS). Table 2 revealed that HFPS contained 92.07 ± 0.57% total sugars, 40.55 ± 0.38% uronic acids, and 0.69 ± 0.03% protein, 5.94 ± 0.08% polyphenol, indicating that most proteins were removed, with only a minor portion tightly bound to the polysaccharide. Zhang et al. eluted polysaccharides from rose petals using deionized water and 0.3 M NaCl, obtaining neutral RRPS-1 and acidic RRPS-2 containing 16.23% uronic acids, respectively (19). Combined with our current chemical composition analysis, it is shown that HFPS is an acidic polysaccharide.

**Table 2:** Chemical compositions of HFPS (%).

Sample	Total sugar	Uronic acids	Protein	Polyphenol
HFPS	92.07 ± 0.57	40.55 ± 0.38	0.69 ± 0.03	5.94 ± 0.08

### 3.2 Monosaccharide composition and Mw distribution

**Figure 2:**
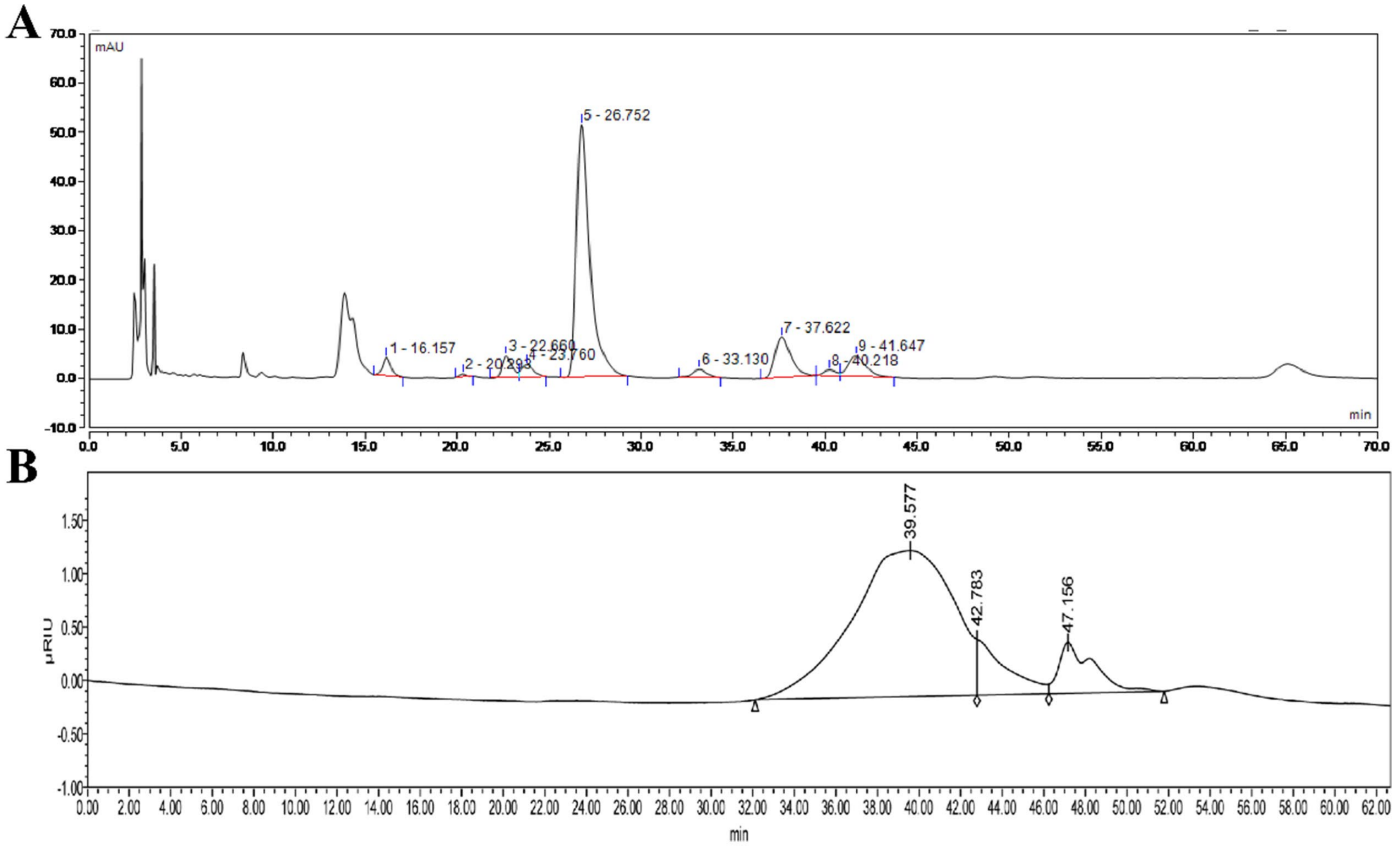
High-performance liquid chromatography of monosaccharide compositions of HFPS **(A)** and HPGPC profiles of HFPS **(B)**.

The monosaccharide composition analysis of HFPS (Figure 2A) revealed the molar ratio of GalA:Gal:Ara:GlcA:Man:Rha:Glc:GlcN was 70.70:14.11:4.08:3.60:3.26:2.70:1.21:0.38, indicating its predominant acidic monosaccharide composition, consistent with the chemical composition analysis. Zhang et al. extracted three pectin polysaccharides (GFPPs) from *gardenia* fruits, including acid-extracted pectin (ACP), hot water-extracted pectin (HWP), and alkali-extracted pectin (ALP), with GalA being the highest proportion ranging from 40.96–52.73% (13). According to their report, it is speculated that the alkali-extracted HFPS may be a pectin polysaccharide. HPGPC profiles of HFPS (Figure 2B) showed that HFPS was heterogeneous (4), comprising two Mw distributions, including 9,178 Da (81.41%, 39.577 min) and 2,456 Da (8.89%, 42.783 min), with the peak around 47.3 min representing the salt peak from the mobile phase. Gao et al. extracted lily polysaccharides using polyethylene glycol-based ultrasound-assisted enzymatic extraction, revealing a monosaccharide composition dominated by Gal, Glc, Rha, and Ara in a molar ratio of 6.36:76.03:2.02:7.09 (20). Zheng et al. found that *Hibiscus sabdariffa L.* polysaccharides are mainly composed of arabinose, xylose, and mannose, with a molar ratio of 1:1.34:15.6 and an average molecular weight of 3.3 × 10^5^ (21), suggests that different extraction methods have a significant effect on polysaccharide composition (22). These results indicate that the flower species and extraction method significantly influence the monosaccharide composition of polysaccharides.

### 3.3 Fourier transform infrared spectrometer (FT-IR) analysis

**Figure 3:**
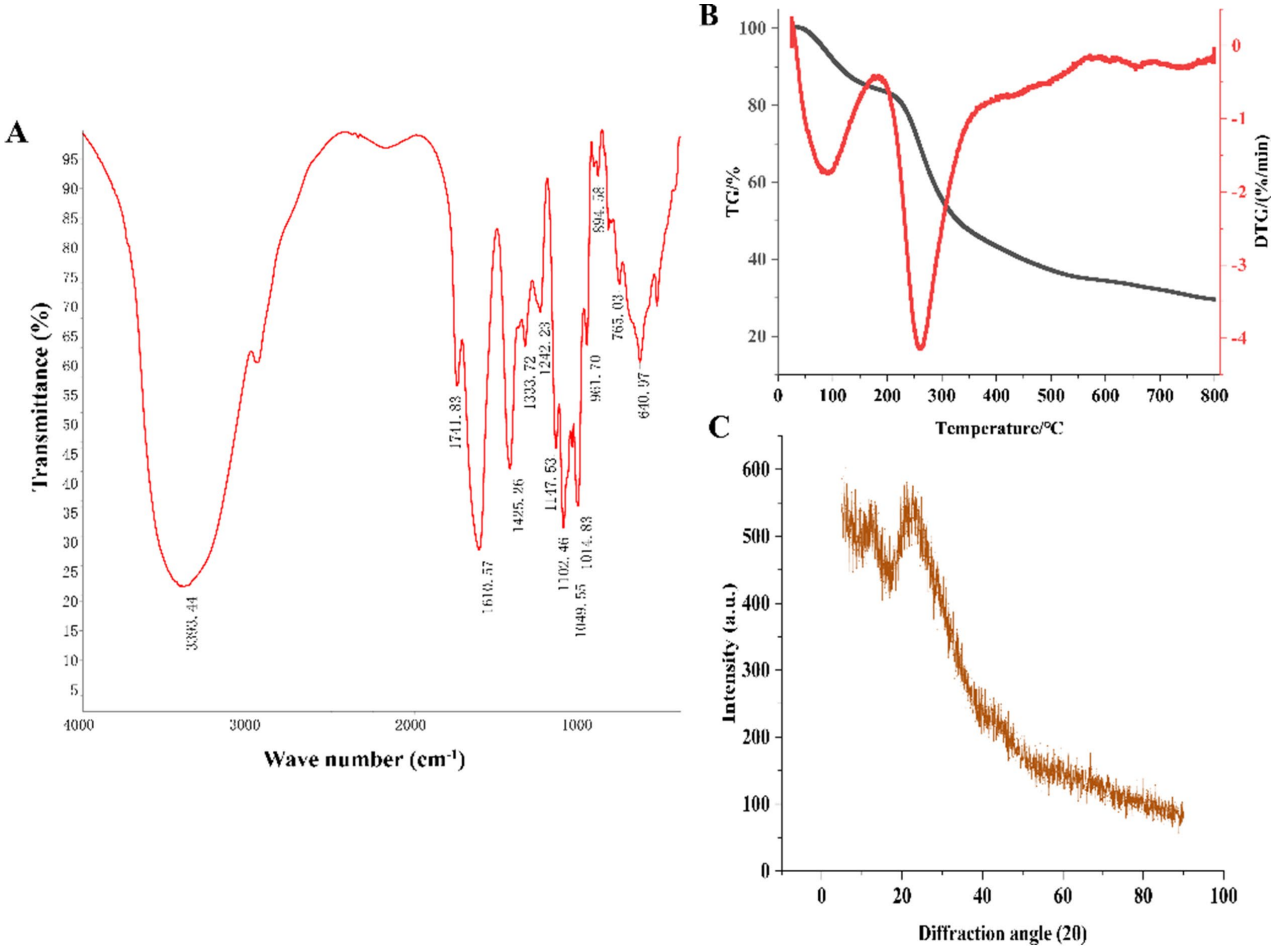
FT-IR **(A)**, TG **(B)** and XRD **(C)** of HFPS.

As shown in Figure 3A, the strong and broad peak at 3,393 cm^−1^ corresponded to the characteristic –OH absorption (23). The peak at 1,741 cm^−1^ was attributed to the C=O stretching vibration of carboxyl groups, consistent with the presence of uronic acids detected in the monosaccharide composition analysis. The absorption peak at 1,610 cm^−1^ was due to the C=O stretching vibration of –CHO groups. The sharp peak at 1,425 cm^−1^ was attributed to the C-H bending vibration, corresponding to the β-glycosidic linkage vibration, a characteristic peak for polysaccharides (24). The peak at 1,333 cm^−1^ represented the C–N stretching vibration. The peak at 1,242 cm^−1^ was attributed to the S=O stretching vibration, indicating the presence of sulfate groups. The absorption peaks at 1,147 cm^−1^, 1,102 cm^−1^, and 1,014 cm^−1^ were caused by the C–O–C and C–O–H stretching vibrations in the pyranose ring, suggesting the presence of pyranose glycosidic linkages in HFPS (25). Hexoses like GalA typically exist in the pyranose form, consistent with the monosaccharide composition analysis. The peak at 961 cm^−1^ was ascribed to the non-symmetric C–O–C skeletal stretching vibration of D-pyranose. The absorption peak at 894 cm^−1^ was characteristic of β-glycosidic linkages, while the peaks in the 800–500 cm^−1^ region indicated the presence of pyranose ring skeletons in the polysaccharide (25).

### 3.4 Thermogravimetry (TG), X-ray diffraction (XRD), scanning electron microscopy (SEM), and nuclear magnetic resonance (NMR) analysis

**Figure 4:**
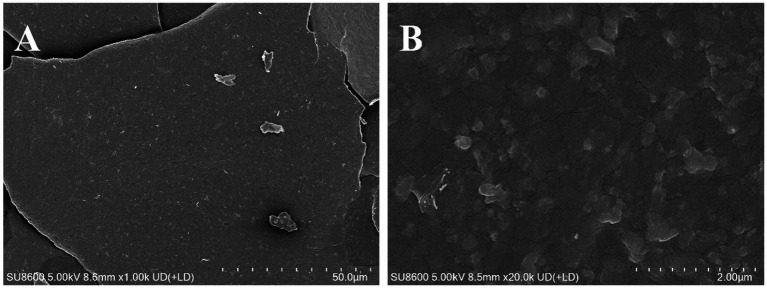
SEM images of HFPS under 1 k× **(A)** and 20 k× **(B)**.

The thermal stability and degradation temperature of HFPS can be obtained from the TG and DTG curves (26). As shown in the TG curve (Figure 3B), HFPS exhibited noticeable weight loss in the temperature ranges of 25–150°C and 200–500°C. The weight loss between 25 and 150°C was relatively minor, likely due to the loss of physically adsorbed water in the polysaccharide, while the substantial weight loss from 200 to 500°C could be attributed to the depolymerization of HFPS and the cleavage of C–O and C–C bonds in the sugar ring units (27). The total weight loss of HFPS was 70.43% in the temperature range of 0–800°C. The DTG curve revealed two temperatures corresponding to the maximum weight loss rates, at 93.06°C and 259.89°C. The XRD analysis (Figure 3C) showed a prominent peak at 25° for HFPS, indicating its propensity for crystallization and the presence of a locally ordered structure, consistent with previous studies reporting diffraction peaks around 20° for various polysaccharides (28, 29). SEM analysis can provide qualitative information about the surface morphology of polysaccharides. As depicted in Figures 4A,B, HFPS exhibited a rough surface with a compact sheet-like arrangement. This porous and lamellar conformation can expose the active sites of the polysaccharide and increase its specific surface area, facilitating its utilization and adsorption, thereby enhancing its bioactivity (29).

**Figure 5:**
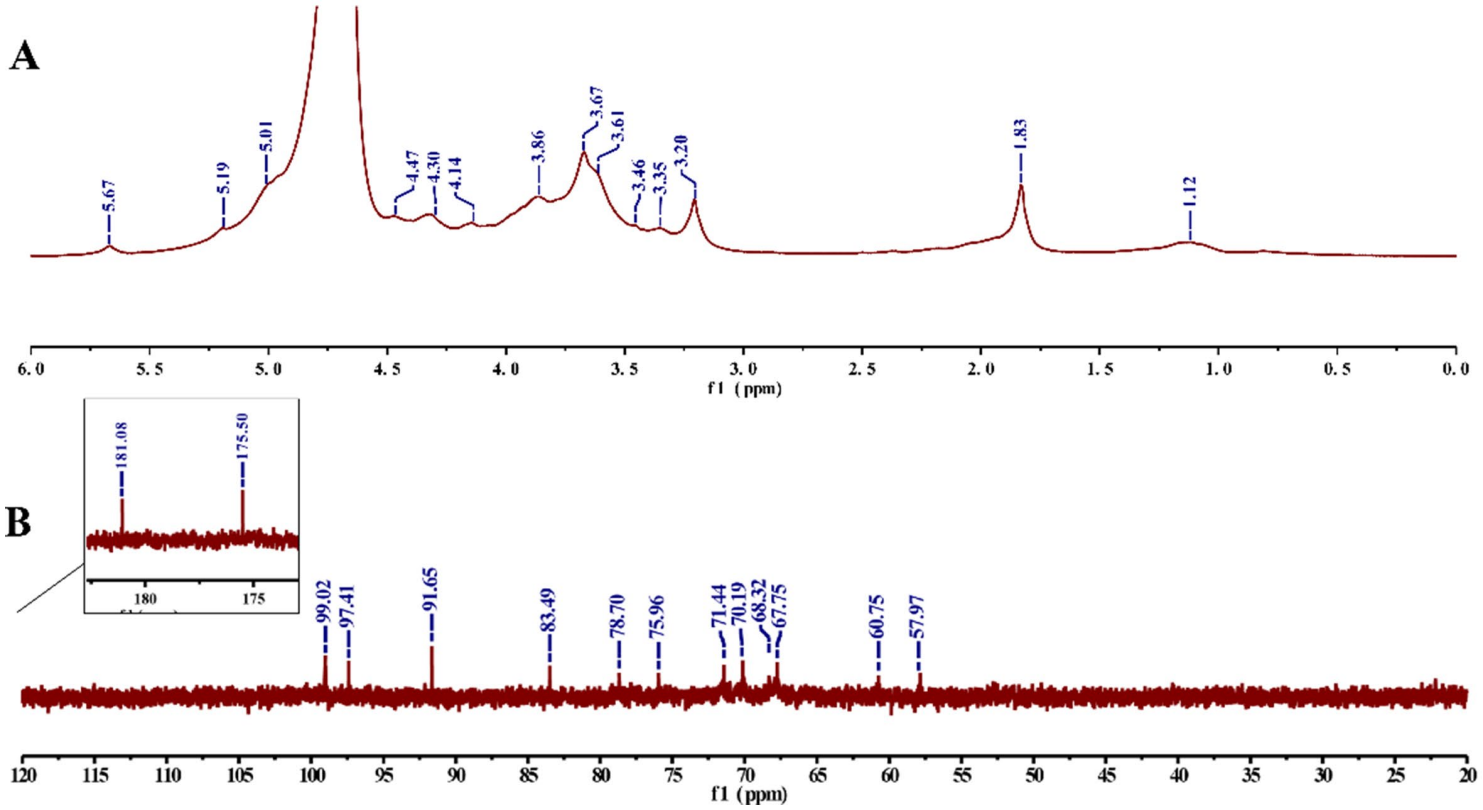
^1^H NMR **(A)** and ^13^C NMR **(B)** spectrum of HFPS.

The NMR spectra (Figure 5) showed that the signals in the *δ* 3.2–4.2 ppm correspond to those of the protons on the polysaccharide glycosyl cyclic carbons (C2–C6), whereas the signals at *δ* 5.01, 5.19, and 5.67 ppm belong to the proton on the polysaccharide heterogeneous carbon (C1) (14), and there was only a weak signal at *δ* 4.47 ppm in the range of δ 4.4–4.9 ppm, derived from β-glycosidic linkages, which matched the peak pattern at 894 cm^−1^ in FT-IR, indicating the dominance of α-glycosidic bonds in HFPS (30). In addition, *δ* 99.02 ppm corresponds to the α-isomerized carbon in the ^13^C NMR spectrum (31), which is consistent with the results of the ^1^H NMR spectrum. *δ* 181.08, 175.50 ppm indicate that HFPS has two distinct uronic acid (32). The C2–C5 signal regions of polysaccharides were distributed at *δ* 67.75–78.7 ppm, whereas the C6 signal region had a distribution of *δ* 60.75, 57.97 ppm. *δ* 5.67 ppm anomalous proton signals and *δ* 99.02 ppm anomalous carbon signals corresponded to (1 → 4)-α-glucose.

### 3.5 Free radical scavenging activity

**Figure 6:**
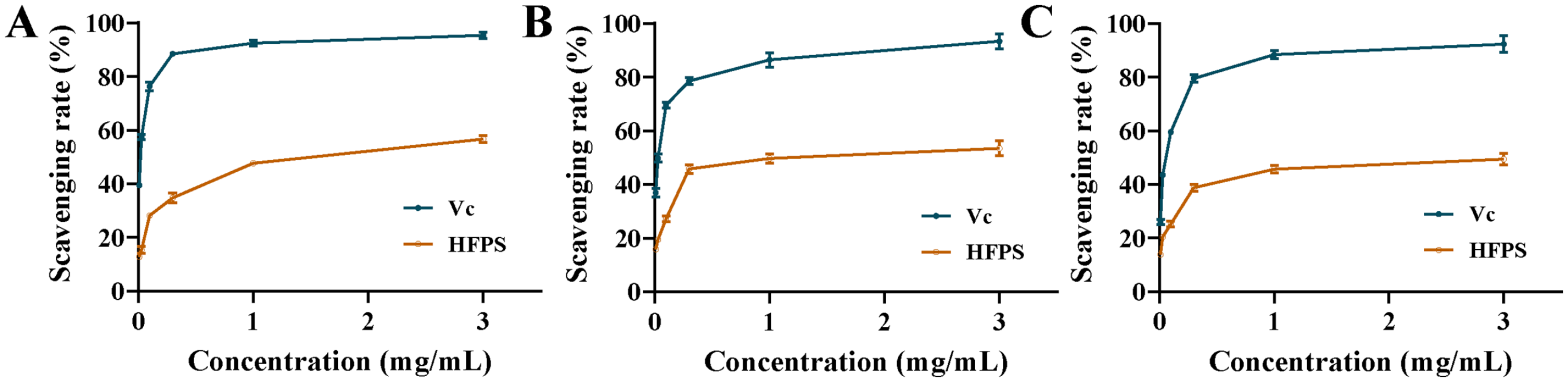
Scavenging capability of HFPS, including ABTS· **(A)**, DPPḤ **(B)**, and ·OH **(C)**.

As illustrated in Figure 6, within the concentration range of 0–3 mg/mL, the scavenging rates against ABTS·, DPPḤ, and ·OH radicals were increased with increasing HFPS concentration, demonstrating a positive correlation between the free radical scavenging capability of HFPS and its concentration within this range. At a concentration of 3 mg/mL, HFPS exhibited scavenging rates of 56.67, 53.46, and 49.49% for ABTS·, DPPḤ, and ·OH radicals, respectively. Similarly, Afshari et al. verified the potent scavenging activity of *Hibiscus sabdariffa* leaf polysaccharides against DPPH and hydroxyl radicals *in vitro* (12). The scavenging effect of HFPS on OḤ was attributed to the high oxidation ability of the free radical. HFPS contains a large amount of uronic acid, and OḤ can selectively capture hydrogen from C-5 of sugar (33). Most studies have shown that relatively good free radical scavenging activity is associated with polysaccharides with lower molecular weight (4). Among these polysaccharides, more reductive-OH terminals can be used to receive and eliminate free radicals (34). The carboxyl group of GalA can react with free radicals, thus effectively reducing oxidative stress (35). This shows that the composition and structure of HFPS play an important role in its antioxidant activity.

### 3.6 Antioxidant activity against oxidative stress

**Figure 7:**
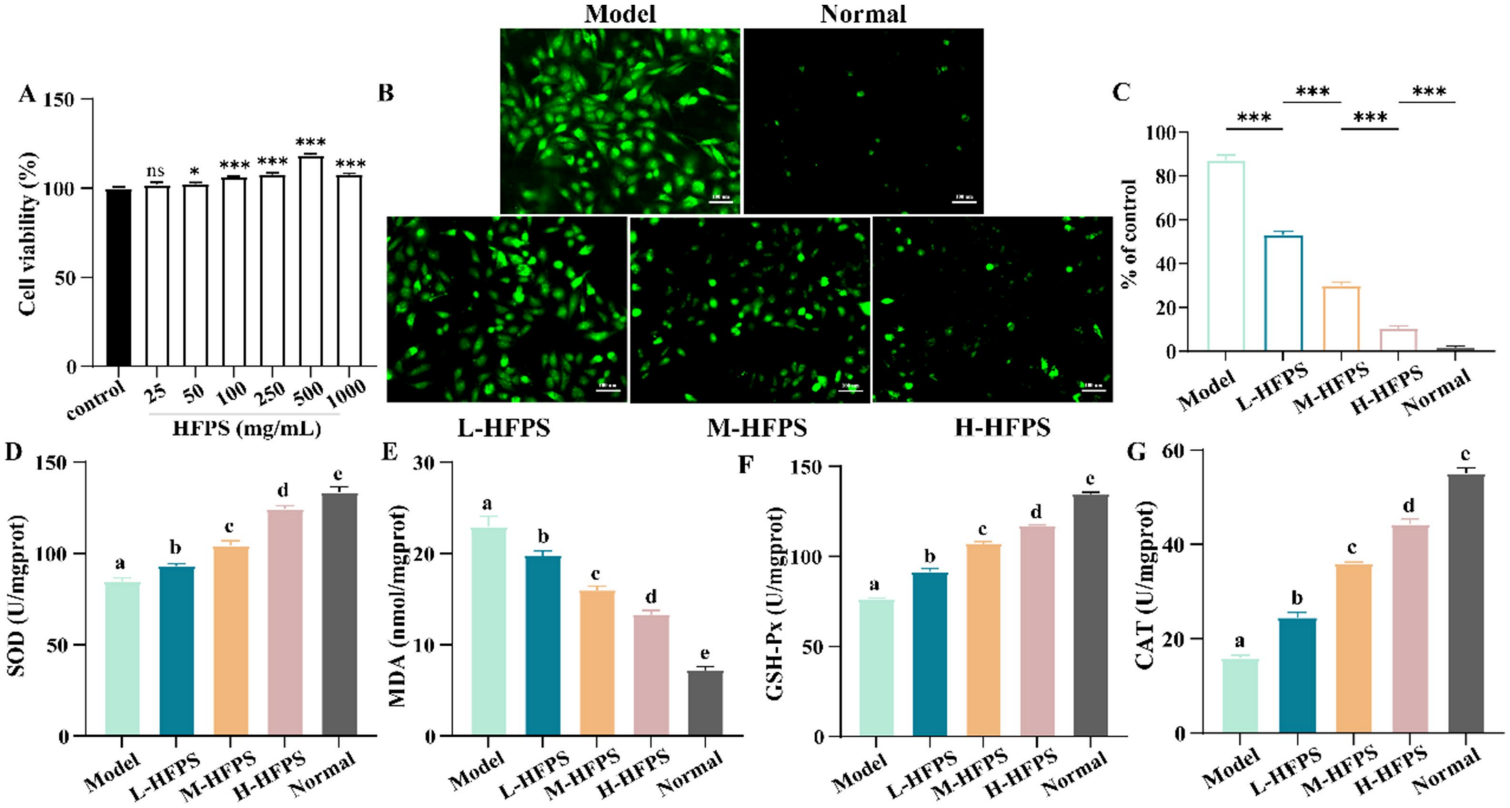
Cell viability of HepG2 cells treated with HFPS **(A)**, ROS distribution in HepG2 cells under a fluorescence microscope **(B,C)**, SOD **(D)**, MDA **(E)**, GSH-Px **(F)**, and CAT **(G)** levels of HepG2 cells treated with HFPS. Different letters represent significant differences between groups, *p* < 0.05; *p < 0.05; **p < 0.01, ***p < 0.001; ****p < 0.0001; ns, not significant.

Hydrogen peroxide (H_2_O_2_), a potent oxidizing agent, can rapidly penetrate cell membranes and damage the structure and function of cellular biomolecules, such as lipids, proteins, and nucleic acids when introduced to cells. Previous studies have successfully established oxidative damage models in various cell types using H_2_O_2_ (36). To investigate the potential mechanisms of HFPS in antioxidant activity, we constructed an H_2_O_2_-induced free radical damage cell model. Initially, we evaluated the cytotoxicity of HFPS on HepG2 cells (Figure 7A), revealing no significant toxicity within the range of 0–1,000 μg/mL, with the highest cell viability observed at 500 μg/mL. Therefore, concentrations of 100, 250, and 500 μg/mL were selected as low, medium, and high doses of HFPS for subsequent experiments. H_2_O_2_ treatment significantly increased the ROS levels in HepG2 cells. However, HFPS supplementation reversed this effect in a dose-dependent manner (Figures 7B,C). Under H_2_O_2_ stimulation, excessive ROS production disrupts cellular defense mechanisms and triggers oxidative stress, leading to irreversible mitochondrial damage and impairment of cellular structure and function (37). Recent studies have shown that acidic polysaccharides from ginseng exhibit better ROS scavenging ability compared to neutral polysaccharides, potentially due to their higher uronic acid content (38).

Intracellular antioxidant enzymes, such as superoxide dismutase (SOD) and glutathione peroxidase (GSH-Px), promptly eliminate reactive oxygen species, free radicals, and other oxidative products generated during metabolism (39). The level of the oxidation product malondialdehyde (MDA) reflects the degree of oxidative damage sustained by cells (40). We measured the levels of oxidative stress-related cellular factors and key enzyme activities, as shown in Figure 7E. H_2_O_2_ treatment led to an increase in MDA levels, which was inhibited by HFPS intervention at different concentrations. Furthermore, HFPS treatment at various concentrations reversed the H_2_O_2_-induced decrease in SOD, catalase (CAT), and GSH-Px activities (Figures 7D,F,G). SOD, CAT, and GSH-Px in cells possess defensive functions against oxidative damage and protect cells from ROS-induced injury (41). SOD catalyzes the dismutation of superoxide anions into oxygen and hydrogen peroxide, CAT decomposes hydrogen peroxide into water and oxygen, and GSH-Px converts toxic peroxides into harmless products through the oxidation of glutathione (18). Consistently, Lin et al. found that polysaccharides from *Cyclocarya paliuru*s enhanced the antioxidant defense system by upregulating the activities of SOD, CAT, and GSH-Px (42). Li et al. reported that an acidic polysaccharide from *Dioscorea opposita* increased SOD activity and reduced ROS production and MDA levels in H_2_O_2_-treated cells (43). The high GalA content may be a major contributor to the antioxidant activity of HFPS (44).

**Figure 8:**
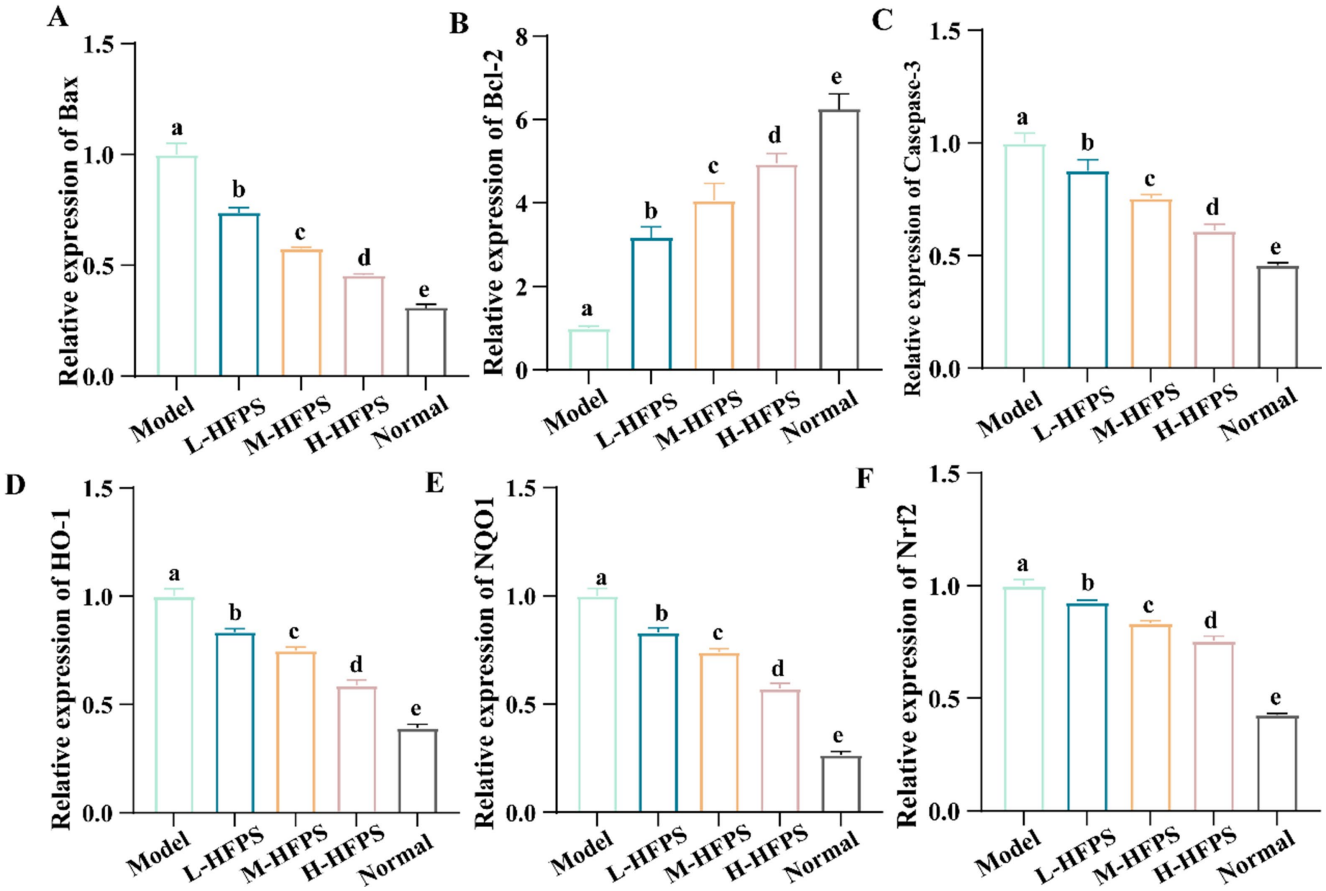
Effects of HFPS on key mRNA expression levels of *Bax* **(A)**, *Bcl*-2 **(B)**, *Casepase-3* **(C)**, *HO-1* **(D)**, *NQO1* **(E)**, *Nrf2* **(F)** in H_2_O_2_-treated HepG2 cells. Different letters represent significant differences between groups, *p* < 0.05.

**Figure 9:**
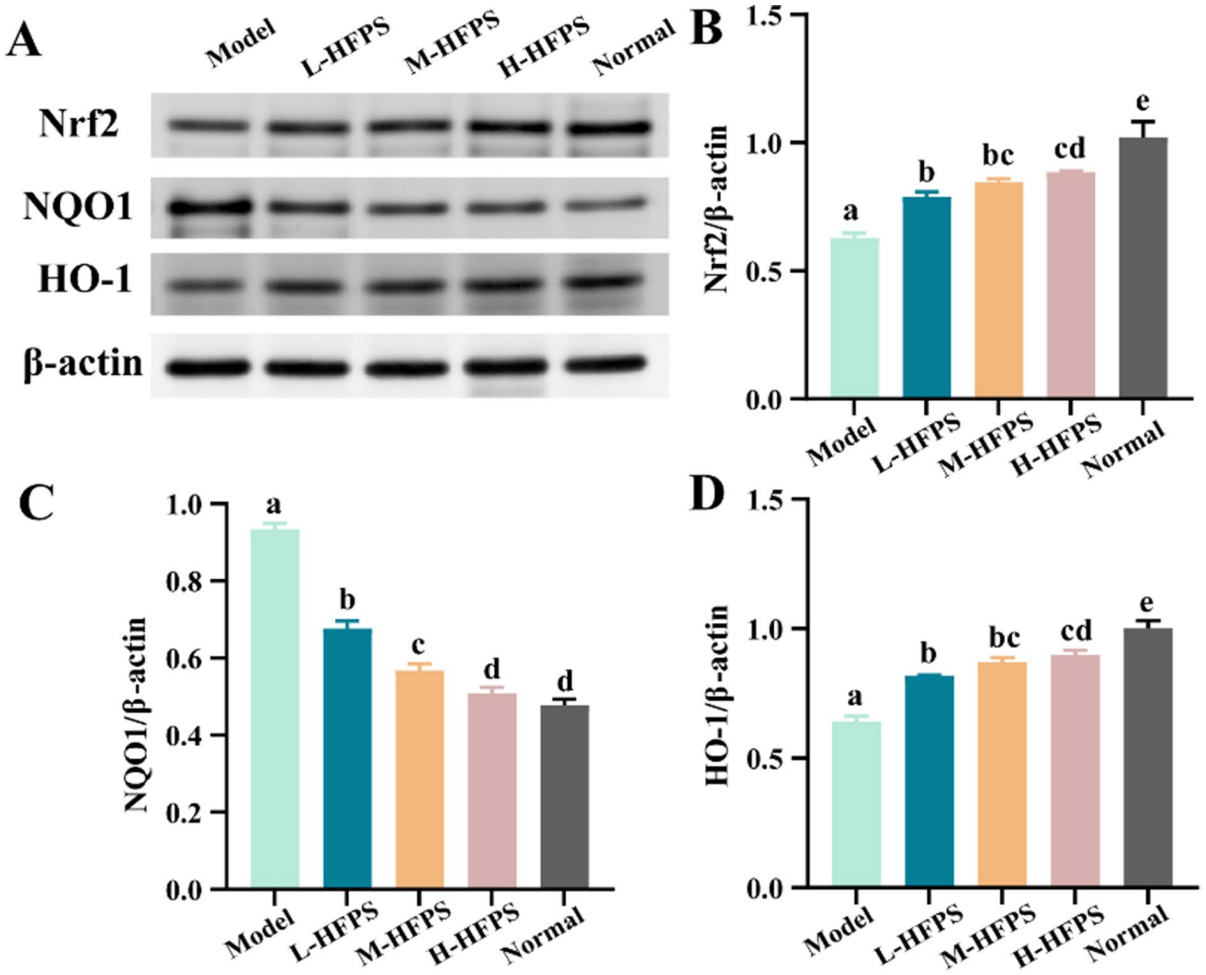
Effects of HFPS on protein expression levels including Nrf2 **(A,B)**, NQO1 **(A,C)**, and HO-1 **(A,D)** in H_2_O_2_-treated HepG2 cells. Different letters represent significant differences between groups, *p* < 0.05.

To further elucidate the antioxidant mechanisms of HFPS, we analyzed its effects on the expression levels of antioxidant-related genes. HFPS reversed the H_2_O_2_-induced increase in *B-cell lymphoma-2* (*Bcl-2*)-*associated X protein* (*Bax*) and *Caspase-3* mRNA levels and upregulated *Bcl-2* expression in a concentration-dependent manner (Figures 8A–C). Bcl-2 is a classical anti-apoptotic protein, and its overexpression can reduce the production of oxygen free radicals and the formation of lipid peroxides. Bax is a crucial pro-apoptotic protein that can form heterodimers with Bcl-2, inhibiting its function (18). Caspase-3 can be cleaved and activated by various proteases, initiating a Caspase-3 cascade reaction that leads to cell apoptosis. These results indicate that HFPS can ameliorate oxidative stress-induced cell apoptosis. Additionally, the mRNA levels of *heme oxygenase-1 (HO-1)*, *NAD (P)H: quinone oxidoreductase 1 (NQO1)*, and *nuclear factor erythroid 2-related factor 2 (Nrf2)* were significantly increased after HFPS treatment (Figures 8D–F). Consistent with the mRNA expression results, HFPS treatment markedly elevated the protein levels of Nrf2 and its downstream targets, NQO1 and HO-1, compared to the Model group (Figure 9). *Taraxacum mongolicum* polysaccharide (TMP) has been shown to enhance the activities of antioxidant enzymes (SOD, CAT, and GPX) and Nrf2 expression (45), suggesting that polysaccharides may be effective modulators of the Nrf2/NF-κB signaling pathway. Nrf2 is a crucial transcription factor that inhibits oxidative stress and inflammatory responses. Under oxidative stress conditions, Nrf2 is a key transcription factor that induces the expression of various antioxidant enzymes, including SOD, HO-1, NQO1, GSH-Px, and CAT (46). These enzymes regulate the redox balance within the body, allowing it to recover from oxidative stress to a normal physiological state. HO-1 is an Nrf2-regulated antioxidant protein that catalyzes the degradation of the potent oxidant heme, with the degradation products exhibiting strong antioxidant effects by scavenging ROS and oxygen free radicals (47). The activation of the Nrf2/HO-1 pathway exerts a negative regulatory effect on intracellular oxidative reactions (48). The carboxylic and phenolic hydroxyl groups in HFPS provide good antioxidant capacity. These groups can neutralize oxidants by forming hydrogen bonds or reacting with free radicals to reduce oxidative damage to cells. Meng et al. reported that gray mangrove polysaccharides targeted Nrf2 to upregulate HO-1, NQO1, SOD, and GSH-Px, thereby counteracting LPS/D-GalN-induced liver inflammation in mice (46). Recent studies have shown that ginseng polysaccharides significantly promote the production of antioxidant enzymes (SOD, CAT, and GSH-Px) and reduce the levels of oxidative stress markers (ROS and MDA) (49). Furthermore, their antioxidant mechanism may be associated with the Keap1/Nrf2 signaling pathway. Zhang et al. found that floral polyphenols decreased the levels of ROS and MDA and increased the activities of SOD, GSH-Px, and CAT in LPS-treated SH-SY5Y cells (50). However, there are no studies on the antioxidant activity of floral polysaccharides at the cellular level. Therefore, we propose that HFPS can modulate redox homeostasis through the Nrf2/HO-1 pathway, reduce H_2_O_2_-induced ROS accumulation, and improve the activities of oxidative stress-related enzymes, thereby exerting its antioxidant effects.

## 4 Conclusion

In this study, a novel pectin polysaccharide (HFPS) was successfully isolated and purified from the flowers of *Hibiscus syriacus* Linn., and demonstrated significant antioxidant activities. Notably, HFPS exhibited a high galacturonic acid content, which is crucial for its antioxidant properties. Structural analysis revealed the presence of α-glycosidic linkages, a semi-crystalline structure, and a porous, lamellar surface morphology, which could enhance its bioavailability. Notably, HFPS exhibited significant free radical scavenging activities and effectively protected HepG2 cells against H_2_O_2_-induced oxidative stress by reducing ROS accumulation, modulating redox-related enzymes, and alleviating oxidative damage. Mechanistic investigations revealed that HFPS could upregulate the expression of antioxidant-related genes, including *Bcl-2*, *HO-1*, *NQO1*, and the crucial transcription factor *Nrf2*, while downregulating pro-apoptotic genes like Bax and Caspase-3, suggesting its antioxidant effects are mediated by modulating the Nrf2/HO-1 pathway and redox homeostasis. In conclusion, the present study elucidated the structural features and antioxidant mechanism of HFPS, which can be used in the future to systematically analyze the effects of different sources and structures of *Hibiscus* polysaccharides on antioxidant activity and to identify the key structural features, which can guide the development of more effective functional products in the direction of natural antioxidants and nutritional supplements.

## Data Availability

The raw data supporting the conclusions of this article will be made available by the authors without undue reservation.
